# Desensitizing Addiction: Using Eye Movements to Reduce the Intensity of Substance-Related Mental Imagery and Craving

**DOI:** 10.3389/fpsyt.2016.00014

**Published:** 2016-02-08

**Authors:** Marianne Littel, Marcel A. van den Hout, Iris M. Engelhard

**Affiliations:** ^1^Clinical Psychology, Utrecht University, Utrecht, Netherlands

**Keywords:** EMDR, eye movements, addiction, food craving, cigarette craving, working memory taxation, mental imagery, addiction memory

## Abstract

Eye movement desensitization and reprocessing (EMDR) is an effective treatment for posttraumatic stress disorder. During this treatment, patients recall traumatic memories while making horizontal eye movements (EM). Studies have shown that EM not only desensitize negative memories but also positive memories and imagined events. Substance use behavior and craving are maintained by maladaptive memory associations and visual imagery. Preliminary findings have indicated that these mental images can be desensitized by EMDR techniques. We conducted two proof-of-principle studies to investigate whether EM can reduce the sensory richness of substance-related mental representations and accompanying craving levels. We investigated the effects of EM on (1) vividness of food-related mental imagery and food craving in dieting and non-dieting students and (2) vividness of recent smoking-related memories and cigarette craving in daily smokers. In both experiments, participants recalled the images while making EM or keeping eyes stationary. Image vividness and emotionality, image-specific craving and general craving were measured before and after the intervention. As a behavioral outcome measure, participants in study 1 were offered a snack choice at the end of the experiment. Results of both experiments showed that image vividness and craving increased in the control condition but remained stable or decreased after the EM intervention. EM additionally reduced image emotionality (experiment 2) and affected behavior (experiment 1): participants in the EM condition were more inclined to choose healthy over unhealthy snack options. In conclusion, these data suggest that EM can be used to reduce intensity of substance-related imagery and craving. Although long-term effects are yet to be demonstrated, the current studies suggest that EM might be a useful technique in addiction treatment.

## Introduction

Eye movement desensitization and reprocessing (EMDR) is a well-established, effective treatment for posttraumatic stress disorder [PTSD: (1, 2)]. During EMDR, patients recall their traumatic memories while making horizontal eye movements (EM). This decreases the sensory richness of the memories and makes them less emotionally intense. Interestingly, mounting research shows that EM can also decrease the vividness and emotionality of positively laden memories (3, 4), and images of possible future events (flash-forwards) (5–8). This suggests that EMDR might be suitable for the treatment of other types of psychopathology in which maladaptive memory and mental imagery plays a role, including addictive disorders (9).

Addictive disorders are chronic and relapsing in nature and pose a widespread problem with great societal, economic, and personal costs. Remission rates are extremely high, with more than 85% of individuals returning to substance use within 1 year after quitting (10). Over the past years, there has been little progress in identifying new, effective interventions, and relatively few existing interventions have been validated experimentally (11). The present studies were designed to provide proof-of-principle for the use of EMDR in the treatment of addiction. More specifically, it was examined whether making EM during the recall of substance-related images can reduce their vividness, emotionality, and ability to elicit craving, as well as general craving and substance-use behavior.

Eye movement desensitization and reprocessing was originally developed by Shapiro (12) to facilitate the cognitive processing of traumatic memories. In the basic EMDR protocol (13), the client is instructed to hold an unpleasant memory in mind, while EM is induced by having the client follow a side-to-side motion of the therapist’s index finger. The client then reports current sensations, cognitions, and emotions, including the distress caused by the memory. Sets of EM are repeated until the client reports that the distress has been reduced to a minimal level. Then, the client is guided to practice a positive cognition to go with the memory. Multiple meta-analyses show that EMDR is effective in the treatment of PTSD (1, 2, 14). Practice guidelines now consider both cognitive behavior therapy (CBT) and EMDR to be treatment of choice. Importantly, a meta-analysis by Lee and Cuijpers (15) shows that the EM component of the therapy has significant additional value over and above repeated activation of the memory without EM. In addition, numerous lab studies [e.g., Ref. (16, 17); and also see Ref. (18) for an overview] show that autobiographical memories become less vivid and emotional after applying only the EM component of EMDR, as compared to memory recall only. Hence, EM seems important for EMDR to have its effects, but it is still unclear how this works.

A plausible explanation of the effects of EM is provided by the working memory (WM) theory. WM is a cognitive system for temporary storage and manipulation of information (19, 20) and has limited capacity. During EMDR, people simultaneously recall traumatic memories and make EM, two processes that have both been demonstrated to tax WM (17, 21). The subsequent competition for its limited capacity affects memory recall. Memories are processed in a more detached manner and become less vivid and emotional. This memory “blurring” does not only take place during or immediately after the intervention but also appears to have long-term effects [i.e., 1 day or week later; (16, 22)]. EMDR seems to exploit the fact that the retrieval of memories returns them to a labile state, during which they can be altered or updated (23, 24). After memory recall plus EM, less vivid, less emotional, and less detailed versions of memories are reconsolidated into long-term storage.

Evidence for the WM theory of EMDR is provided by many well-controlled lab studies. They show that simultaneous EM reduce memory vividness, but so do other dual WM tasks, such as mental arithmetic (7) or copying a complex drawing (22), compared to memory recall without a dual task. Furthermore, and as noted before, negative memories are affected by dual WM tasks, but so are other kinds of taxing mental images, including positive memories [e.g., Ref. (3–5)] and distressing images about possible future events (flash-forwards) (3, 5–8).

In addictive disorders, the retrieval of substance-related memories is crucial to the experience of craving, which is, in turn, a strong predictor of substance use maintenance and relapse (25–27). These substance-related memories include classically and instrumentally learned associations between cues and effects (e.g., the association between feeling stressed and smoking and between smoking and becoming relaxed). They also include episodic memories, such as memories of specific encounters with the substance (e.g., a great first use experience), memories of substance use consequences, and memories of loss of self-control and relapse (9, 28). Craving is often maintained and augmented by sensory imagery [e.g., imagining sight, smell, future use: (29, 30)]. Research shows that instructions to form mental images of substance use increase craving [e.g., Ref. (31, 32)], with more vivid imagery predicting higher craving intensity (31, 33–35).

Craving can be reduced by dual task procedures. Many studies have shown that engaging in non-substance-related imagery or visuospatial tasks while experiencing high craving levels reduces craving frequency and intensity [for overviews, see Ref. (36, 37)]. Concurrent cognitive activity therefore provides a valuable way of coping with the acute effects of craving and can be easily implemented in clinical practice [e.g., Ref. (38)]. When craving is experienced, one can engage in a dual task. However, this method requires substance-dependent persons to identify craving while it can still be controlled, whereas self-monitoring, self-evaluation, and cognitive control are often compromised in addiction (39). Furthermore, and in contrast to EMDR, this method is not designed to alter substance-related representations in memory storage, and long-term effects are not expected after one quits using it. To achieve prolonged craving reduction, specific instructions must be given to retrieve the images before engaging in the dual task. Only reactivated memories enter a labile state and are susceptible to alteration or disruption (23, 24).

Three studies so far have investigated the effects of visuospatial WM tasks (33, 40, 41) during *instructed* imagery of favorite foods in a sample of healthy (non-preselected) students. All tasks significantly reduced the vividness of the food-related imagery and craving compared to a control condition. Although long-term effects were not measured, these studies provide first indications that concurrent tasks can degrade substance-related images.

Research on the effectiveness of the full EMDR procedure in addiction is limited. In most studies, EMDR predominantly focused on traumatic memories constituting comorbid PTSD and not on memory representations or sensory imagery constituting substance craving and dependence itself (42). The investigations of EMDR that did specifically target substance-related memories are clinical anecdotes or case reports [for a list, see Ref. (43)]. Although most of them describe positive results, some found mixed (44) or negative results (45). Only one controlled study has been published so far (46). In this study, thirty alcohol-dependent patients received either treatment as usual (TAU) along with two EMDR sessions or TAU only. Target memories were memories of specific instances of intense craving and relapse. Patients in the TAU + EMDR group showed a significant reduction in alcohol craving one as well as six months posttreatment, compared to patients receiving TAU only. In addition, fewer patients from the TAU + EMDR group relapsed. Unfortunately, the study has several limitations, including small sample sizes and multiple drop-outs on follow-up measures. Nonetheless, the results are encouraging for the application of EMDR targeting specific addiction memories, especially because the effects were obtained after only two sessions.

In order to determine whether EMDR can serve as a promising adjunct to current treatment options for addiction, more research is necessary, including well-controlled proof-of-principle studies showing that EM can desensitize addiction-relevant memory representations and imagery. In the present studies, the effects of EM on the vividness and emotionality of substance-related images and associated craving were investigated. Because craving is triggered by addiction memories and exacerbated by mental imagery, both were used as targets in each of the two studies. In the first study, EM targeted food-related *imagery* and food craving in healthy dieting and non-dieting participants. It extends the studies by Kemps et al. (33), McClelland et al. (40), and Steel et al. (41), by placing more emphasis on the retrieval or formation of food-related mental images before the dual task was introduced. Moreover, our study solely focused on the effects of EM as dual task to reduce craving. Furthermore, there were methodological differences, such as the use of a between-subjects design, which prevents possible carry-over effects of interventions on craving. The second study was concerned with smoking-related *memories* and cigarette craving and was conducted in smokers. Both studies employed the EMDR lab model [cf., Ref. (3, 5, 47)], in which half of the participants recalled a substance-related image while making EM (recall + EM), whereas the other half of the participants recalled the image while keeping eyes stationary (RO). Image vividness, emotionality, and craving were measured before (pretest) and after the intervention (posttest). We expected that recall + EM, relative to RO, would decrease image vividness, emotionality, and craving from pre- to posttest.

## Study 1: The Effects of EM on Food-Related Imagery and Food Craving

The first study focused on craving for food. Although food craving is commonly experienced and plays a significant evolutionary role (48), it is associated with unfavorable outcomes, including high-calorie food consumption and body mass index (BMI) (49), binge eating (50), development of obesity (51), and having difficulty in maintaining a diet (52). Many lines of research demonstrate that parallels exist between drug and food cravings in neuroanatomy, neurochemistry, and learning (53–55), providing the rationale for study 1.

Dieting and non-dieting participants were instructed to actively imagine eating their favorite food. We compared the effects of recall + EM versus RO on the vividness and emotionality of these food-related images, as well as specific craving in response to these images and more general craving for their favorite food. Furthermore, we compared snack choice at the end of the task. It was expected that, compared to RO, recall + EM would decrease craving, vividness, and emotionality of the food-related imagery. We also expected healthier snack choices after EM than RO. Because dieters are trying to exert control over their food intake, they are likely to experience motivational conflict when they think of their favorite food (56). Therefore, we expected that food-related imagery would be more taxing for dieters, resulting in greater effects of the intervention in this group. Generalizability of effects was explored by comparing craving for two other favorite foods at pre- and posttest.

Both the present study and study 2 were approved by the local ethics committee of the Faculty of Behavioral and Social Sciences of Utrecht University. All participants provided written informed consent.

### Methods

#### Participants

Eighty-nine female students (*M* age = 21.5, SD = 2.2) participated in experiment 1. They were recruited *via* advertisements at Utrecht University, specifically calling for non-dieters and dieters. Dieters (*n* = 42) were eligible if they reported to be on a diet with the goal of losing weight. They were on the diet for 3.2 months (SD = 4.4) on average. Individuals with explicit knowledge of EMDR were excluded. Participants received either financial compensation or course credit for participation.

### Materials

#### Eye Movement Task

An EM task [cf., Ref. (3, 5)] was used to simulate the EM component of EMDR. A white dot was presented on a black screen, which moved from side-to-side with 1 s per cycle, or a blank screen was presented. The moving dot and blank screens were displayed during four intervals of 24 s separated by 10 s breaks. Participants sat at a 50 cm distance from the computer screen. Participants recalled their food-related image while tracking the dot (recall + EM) or watching the blank screen (eyes stationary; RO).

#### Visual Analog Scales

Before (pretest) and after (posttest) the EM task, participants recalled their food-related images and rated them on vividness using 10 cm Visual Analog Scales (VASs) ranging from 0 (not vivid) to 100 (very vivid), on emotionality using a VAS ranging from 0 (very unpleasant) to 100 (very pleasant), and on image-specific craving (“How strong is your urge to eat [targetfood] at this very moment”) using a VAS ranging from 0 (no craving) to 100 (intense craving). The EM task and VASs were presented using OpenSesame v.0.27.1 (57).

#### General State Food Cravings Questionnaire

Current craving for the target food was assessed with the Dutch translation of the General State Food Cravings Questionnaire [G-FCQ-S: (58)]. This questionnaire consists of 15 items (e.g., “I know I’m going to keep on thinking about tasty [food] until I actually have it”) that are scored on 5-point Likert scales, ranging from “I totally disagree” to “I totally agree.” The reliability is excellent (Cronbach’s α = 0.93). For the purpose of this study, the word “food” was replaced with the participants’ favorite food.

#### General Trait Food Cravings Questionnaire

The General Trait Food Cravings Questionnaire [G-FCQ-T: (58)] was used to measure trait craving, i.e., the tendency to experience craving for food in general. It is composed of 21 questions (e.g., “I feel like I have food on my mind all the time”), which are scored on a 6-point Likert scales. The Dutch translation has good validity and reliability (Cronbach’s α = 0.90).

#### Behavioral Task

As a behavioral outcome measure of EM and RO interventions, participants’ snack choice was measured. At the end of the experiment, all participants were offered an apple or a candy bar. They could pick one of these or refuse both. Choosing an apple and refusing a snack were considered healthy choices, whereas choosing the candy bar was considered an unhealthy choice.

### Procedure

Upon arrival, participants were screened for study eligibility. After signing informed consent, participants were asked several questions about their diet and reported their height and weight, in order to calculate their BMI, and filled out the G-FCQ-T. Then, participants were instructed to select three food items that they craved most at that specific moment. These were entered into the software, and intensity of craving for each food was assessed using on-screen VASs. Out of the three selected foods, participants then picked their favorite one, i.e., the food they craved most at that specific moment. This food became the target for the EM or RO intervention, whereas the other two foods did not (non-targets). First, participants filled out the G-FCQ-S, of which the word “food” was replaced with participants’ target food. They were asked to vividly picture this food and imagine its taste and smell as if they were eating it right now. When the image was clear, they rated vividness and emotionality of this image and image-specific craving using VASs. Subsequently, the EM task started. Half of the participants recalled their image while making EM. The other half recalled their image while keeping eyes stationary (RO). Immediately after the recall + EM or RO intervention, target images were again scored on vividness, emotionality, and craving. The two non-target images were also scored on craving. Then, the G-FCQ-S was filled out for a second time. After finishing this questionnaire, participants proceeded to the behavioral task and were offered the choice between a candy bar and an apple. Participant assignment to recall + EM or RO was counterbalanced. At the end of the experiment, participants were debriefed and given their reward.

### Design and Statistical Analyses

A 2 × 2 × 2 crossed design was used with group (2; dieters, non-dieters) and condition (2; recall + EM, RO) as between-subjects factors and time (2; pretest, posttest) as within-subjects factor.

Five 2 (group) × 2 (condition) × 2 (time) mixed model ANOVAs were conducted to assess whether food-related image vividness, emotionality, and craving VAS scores, G-FCQ-S scores, and non-target craving scores were more reduced after recall + EM than after RO. A Chi-square goodness of fit test was performed to assess whether the healthy snack option would be selected more frequently than would be expected by chance after EM [cf., Ref. (59)]. An alpha level of 0.05 was used for all statistical tests. When the direction of the differences was predicted, one-tailed *p*-values are reported.

### Results

Dieters had significantly higher BMIs (*M* = 23.7, SD = 4.1) than non-dieters (*M* = 21.4, SD = 2.4), *t*(87) = 3.12, *p* < 0.01, and showed greater trait craving (*M* = 72.2, SD = 11.8) than non-dieters (*M* = 64.6, SD = 11.8), *t*(87) = 3.06, *p* < 0.01, indicating that two distinct groups were recruited.

#### Vividness VAS

**Figure 1:**
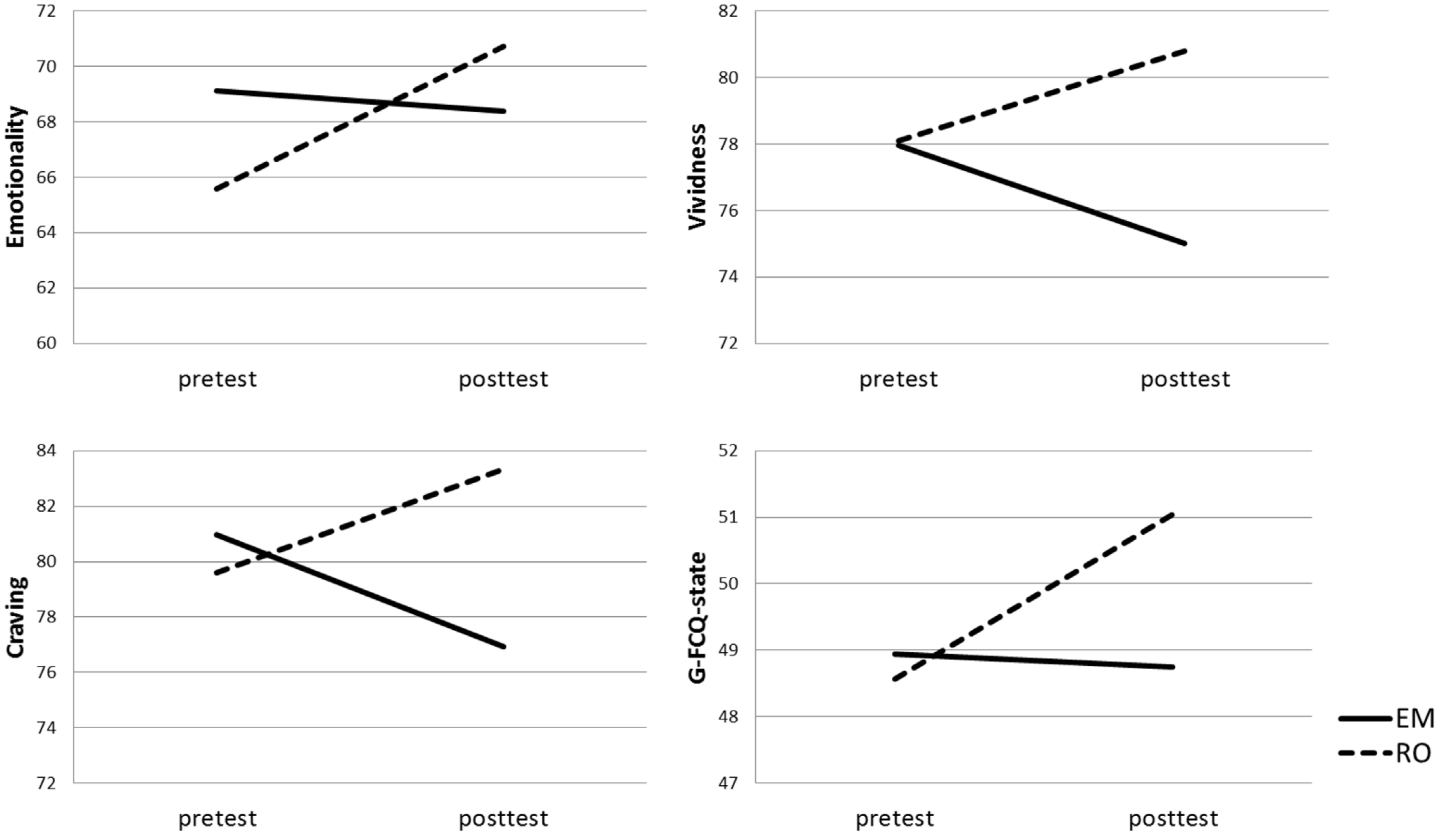
**Mean food craving, emotionality, vividness, and General State Food Craving Questionnaire (G-FCQ-S) scores per Condition [recall + eye movements (EM), recall only (RO)] at pre- and posttest**.

Findings are graphically depicted in Figure 1. There were no main effects of Time, *F*(1,85) = 0.00, *p* = 1, Condition, *F*(1,85) = 0.75, *p* = 0.39, or Group, *F*(1,85) = 1.09, *p* = 0.30. The crucial Condition × Time interaction was significant, *F*(1,85) = 4.01, *p* = 0.05, η^2^ = 0.05. For the RO condition, a significant increase was observed between the pre- and posttest vividness scores, *t*(43) = 1.69, *p* = 0.05, *d* = 0.52. For EM, there was a non-significant trend toward a decrease instead, *t*(44) = 1.33, *p* = 0.10, *d* = 0.41. The Condition × Time interaction effect was not moderated by dieting Group, *F*(1,85) = 0.68, *p* = 0.41.

#### Emotionality VAS

There were no significant main or interaction effects, all *F*’s < 2.36, all *p*’s > 0.13.

#### Craving VAS Target Food

There were no significant main effects of Time, *F*(1,85) = 0.00, *p* = 0.96; Condition, *F*(1,85) = 0.63, *p* = 0.43; or Group, *F*(1,85) = 2.44, *p* = 0.12. However, the crucial Condition × Time interaction was significant, *F*(1,85) = 4.14, *p* = 0.05, η^2^ = 0.05. Paired sample *t*-tests showed that there was a trend for increasing pre- to posttest craving scores in the RO condition, *t*(43) = 1.38, *p* = 0.09, *d* = 0.42, whereas for recall with EM, craving scores dropped significantly from pre- to posttest, *t*(44) = 1.66, *p* = 0.05, *d* = 0.51.

There was a trend for a Condition × Time × Group interaction, *F*(1,85) = 3.32, *p* = 0.07, η^2^ = 0.04. The non-dieting group showed a pre- to posttest increase in craving in the RO condition, *t*(22) = 2.35, *p* = 0.01, *d* = 0.70, and a craving decrease in the EM condition, *t*(23) = 1.90, *p* = 0.04, *d* = 0.57. Craving did not increase or decrease in response to RO or EM in the dieting group, all *t*’s < 0.16, all *p*’s > 0.44.

#### Craving VAS Non-Target Food

A significant main effect of Time was observed, *F*(1,85) = 31.20, *p* < 0.001, indicating a decrease of craving for non-preferred, non-targeted foods over time. Overall, dieters showed significantly less craving in response to their non-preferred foods, *F*(1,85) = 3.99, *p* < 0.05. No other significant effects were found, all *F*’s < 0.36, all *p*’s > 0.55.

#### G-FCQ-State

There was a trend toward a main effect of Time, *F*(1,85) = 2.97, *p* = 0.09, indicating a slight increase of state food craving over time across groups. There were no significant main effects for Condition, *F*(1,85) = 0.76, *p* = 0.58; or Group, *F*(1,85) = 0.09, *p* = 0.76. The crucial Condition × Time interaction was significant, *F*(1,85) = 4.15, *p* = 0.05, η^2^ = 0.05. Paired sample *t*-test showed that for the RO condition, G-FCQ-S scores significantly increased from pre- to posttest, *t*(43) = 3.31, *p* < 0.01, *d* = 0.71, whereas in the EM condition G-FCQ-S scores remained stable over time, *t*(44) = 0.18, *p* = 0.43, *d* = 0.04. There was no significant Condition × Time × Group interaction, *F*(1,85) = 0.01, *p* = 0.91.

#### Snack Choice

**Figure 2:**
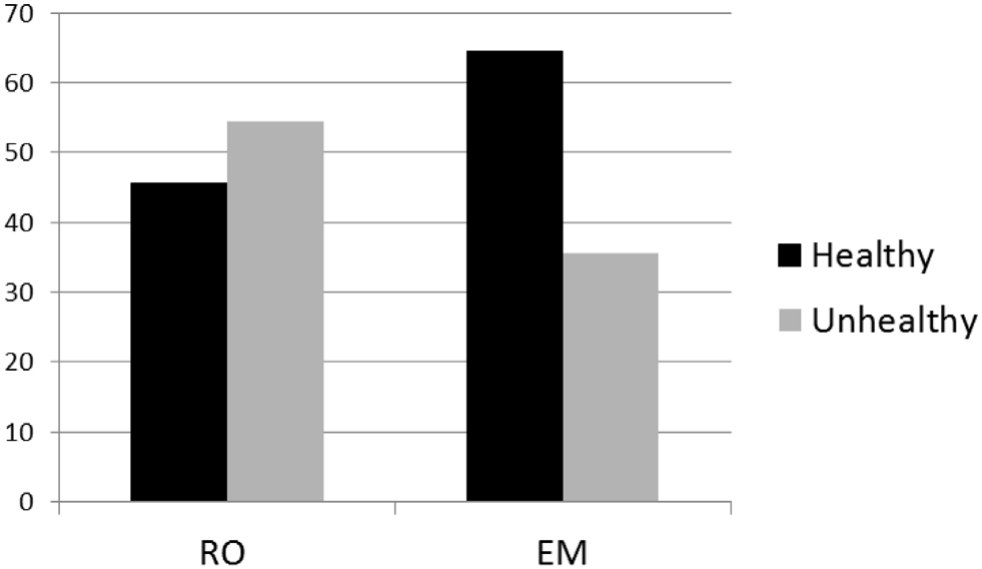
**Percentages of healthy and unhealthy snack choices after recall + eye movements (EM) and recall only (RO) interventions**.

Results are shown in Figure 2. After RO, the frequency of healthy snack choices did not differ from chance, χ^2^ (1) = 0.36, *p* = 0.55. However, after recall + EM, the healthy snack option was more frequently chosen than would be expected by chance alone, χ^2^ (1) = 3.76, *p* = 0.05.

### Discussion

A brief session of EM significantly reduced craving evoked by food-related images compared to a control condition in which no EM were made. This effect was most pronounced in non-dieting participants. In addition, there was a trend for recall + EM to decrease food image vividness, whereas it increased after recalling the image without making EM. General craving for the selected food (G-FCQ-S) did not decrease after recall + EM, but remained stable over time. Note that general craving for food increased after RO, which can be expected due to the passage of time (60) and repeated craving imagery (31, 32). Accordingly, one might argue that making EM during recall attenuates craving. After only a brief application of EM (4 × 24 s), we consider this a clinically relevant result, especially because the G-FCQ-S is a broad measure that incorporates items that do not specifically refer to the preferred food (e.g., “I’m hungry”). Finally, a brief session of EM during food-related imagery affected subsequent snack choice; participants in the EM condition chose the healthier options more often than expected by chance, whereas participants in the RO condition did not.

## Study 2: The Effects of EM on Smoking-Related Memories and Cigarette Craving

In study 2, we compared the effects of recall + EM versus RO on the vividness and emotionality of smoking-related memories, memory-related cigarette craving, and general cigarette craving in daily smokers. In contrast to study 1, RO and EM interventions targeted memories^1^ instead of mental images formed in the lab. Moreover, we presented EM in six sets of 24 s instead of four in order to increase WM taxation [cf., Ref. (8)], and we used a small craving manipulation at the start of the experiment in order to increase craving. Furthermore, because cravings are emotionally ambivalent and likely to involve both positive and negative affect (61), we changed the positive endpoint of the emotionality scale to a neutral one (see Methods). We expected that, compared to RO, recall + EM would decrease craving and the vividness and emotionality of smoking-related memories.

### Methods

#### Participants

Fifty smokers (*M* age = 23.4, SD = 6.6, 58% females, 42% males) participated in experiment 2. They were recruited *via* advertisements at Utrecht University and word-of-mouth and were eligible if they smoked at least five cigarettes per day for 7 days per week. On average, they smoked 10.4 cigarettes per day (SD = 5.8) had smoked for 6.5 years (SD = 6.5). Their mean nicotine dependence level, as measured with the Fagerström test for nicotine dependence (FTND), was 2.0 (SD = 1.9), which can be considered low. They had not smoked for 4.2 h (SD = 4.8) prior to the experiment. Participants received either financial compensation or course credit for participation.

##### EM Task

The EM task was similar to the task used in experiment 1, except that we presented horizontally moving white dots or blank screens during six intervals of 24 s. Participants recalled the image of their smoking-related memory while either tracking the dot or watching the blank screen.

##### Visual Analog Scales

Similar to experiment 1, participants rated their smoking-related memories on vividness and memory-specific craving using 10 cm VASs ranging from 0 (not vivid/no craving) to 100 (very vivid/intense craving) before (pretest) and after (posttest) the EM task. Emotionality was now measured on a 10-cm VAS ranging from not emotional to very emotional.

##### Fagerström Test for Nicotine Dependence

Nicotine dependence levels were measured with the Dutch translation of the FTND (62, 63). The FTND is composed of six items, has good reliability, and correlates significantly with number of cigarettes smoked per day.

##### QSU-Brief

Upon arrival, during pre- and posttest, cigarette craving was measured with the 10-item of the brief questionnaire on smoking urges [QSU-brief: (64)]. This questionnaire is scored on a 7-point Likert scale and contains items like “All I want right now is a cigarette” and “I am going to smoke as soon as possible.” The Dutch translation was used, which has adequate psychometric properties (65).

### Procedure

Participants were instructed to refrain from smoking for at least 1 h prior to the experiment. As an incentive, participants were told that this would be checked with a breath analyzer. Upon arrival, participants were screened for study eligibility and subjected to a non-invasive CO Ppm estimate utilizing the Bedfont piCO simple Smokerlyzer (Bedfont Scientific, Harrietsham, Engeland, 2011; *M* = 14.6 CO Ppm, SD = 9.7). After providing informed consent, participants recalled a recent memory of a specific situation or an emotional state^2^ in which they experienced craving and smoked a cigarette, for example, a get-together with friends in a bar, or feelings of stress. In line with the Dutch EMDR protocol (66), they were asked to “play” these memories in their minds and make a “screen shot” of the most vivid moment. They had to write down keywords of the resulting image. Participants then sat down behind the computer and filled out on-screen questions about demographics and smoking history, the FTND, and the QSU-brief. Then, they underwent a simple craving induction procedure, in which five smoking-related pictures were shown of people smoking or holding cigarettes and inhaling or exhaling cigarette smoke. These pictures were presented full-screen for 5 s. Afterwards, the QSU-brief was administered for a second time. Then, keywords of the selected smoking-related image were entered into the software, and participants were instructed to recall their specific memory for 10 s and rate it on vividness, emotionality, and craving. Next, the EM task started. Half of the participants recalled their memory while making EM. The other half recalled their memory while keeping eyes stationary (RO). Immediately after the recall + EM or RO intervention, memories were again scored on vividness, emotionality, and craving. Then, the QSU-brief was filled out for a third time. Participant assignment to EM or RO was counterbalanced. At the end of the experiment, participants were debriefed and given their reward.

### Design and Statistical Analyses

A 2 × 2 crossed design was used with condition (2; recall + EM, RO) as between-subjects factors and time (2; pretest, posttest) as within-subjects factor.

Four 2 (condition) × 2 (time) mixed model ANOVAs were conducted to assess whether smoking-related image vividness, emotionality, and craving VAS scores, and QSU-brief scores decreased more after recall + EM than after RO. An alpha level of 0.05 was used for all statistical tests. When the direction of the differences was predicted, one-tailed *p* values are reported.

### Results

The craving manipulation caused a significant increase in ­craving, *t*(49) = 5.37, *p* < 0.001.

#### Vividness VAS

There were no significant main effects of Time, *F*(1,48) = 0.06, *p* = 0.81 or Condition, *F*(1,48) = 1.22, *p* = 0.28. The crucial Condition × Time interaction was significant, *F*(1,46) = 4.76, *p* = 0.03, η^2^ = 0.09. Paired sample *t*-test showed that for the RO condition, vividness scores significantly increased from pre- to posttest, *t*(27) = 1.85, *p* = 0.04, *d* = 0.71, whereas in the EM condition vividness scores remained stable over time, *t*(21) = 1.28, *p* = 0.11, *d* = 0.56.

#### Emotionality VAS

For memory emotionality, there were no significant main effects of Time, *F*(1,48) = 0.79, *p* = 0.38 or Condition, *F*(1,48) = 1.85, *p* = 0.18. The crucial Condition × Time interaction showed a non-significant trend toward significance, *F*(1,48) = 2.80, *p* = 0.10, η^2^ = 0.06. In the RO condition, there were no significant differences between pre- and posttest emotionality scores, *t*(27) = 0.56, *p* = 0.29, *d* = 0.22. For EM, the pre- to posttest emotionality scores showed a significant decrease, *t*(21) = 1.87, *p* = 0.04, *d* = 0.82.

#### Craving VAS

There were no significant main effects of Time, *F*(1,48) = 1.62, *p* = 0.21 or Condition, *F*(1,48) = 0.79, *p* = 0.38. However, a significant Condition × Time interaction was observed, *F*(1,48) = 4.19, *p* = 0.05, η^2^ = 0.08. Craving scores significantly increased in the RO condition, *t*(27) = 2.32, *p* = 0.01, *d* = 0.89, whereas for recall + EM, craving scores remained constant over time, *t*(21) = 0.59, *p* = 0.28, *d* = 0.26.

#### QSU-Brief

There were no significant main or interaction effects, all *F*’s < 0.24, all *p*’s > 0.24.

### Discussion

When WM was not taxed during smoking-related memory recall, both memory vividness and memory-evoked craving increased, which is to be expected due to the passage of time (60) and repeated substance-related imagery (31, 32). Because these significant increases were not observed in the recall + EM condition, it might be concluded that image vividness and craving were attenuated by recall + EM. In addition, there was a trend for recall + EM to decrease the emotional intensity of smoking-related images compared to RO.

## General Discussion

Results of the current studies indicate that brief sets of EM during the recall of substance-related images can decrease (study 1) or attenuate (study 2) the craving that is specifically evoked by these images, can attenuate general craving (study 1), can decrease (study 1) or attenuate (study 2) substance image vividness, can decrease image emotionality (study 2), and affect subsequent behavioral choices (study 1), compared to a control condition of substance-related imagery or memory retrieval without EM.

These results are in line with previous studies where EM significantly decreased the vividness and emotionality of autobiographical memories and flash-forwards (18), with three earlier studies in which visual–spatial tasks during food-related imagery decreased image vividness and craving (33, 40, 41), and one RCT among alcohol-dependent patients where two sessions of EMDR in addition to TAU reduced alcohol craving and relapse (46). We extended these studies by applying the methodologically sound EMDR lab model to investigate the effects of EM on substance-related mental images, with special emphasis on the reactivation of these images prior to the EM task [cf., the EMDR protocol (66)]. In addition, we investigated effects of EM on both sensory imagery and substance-related memory representations, we used a different range of outcome measures, including a behavioral measure in study 1, and we were the first to test effects of recall + EM in smokers.

**Figure 3:**
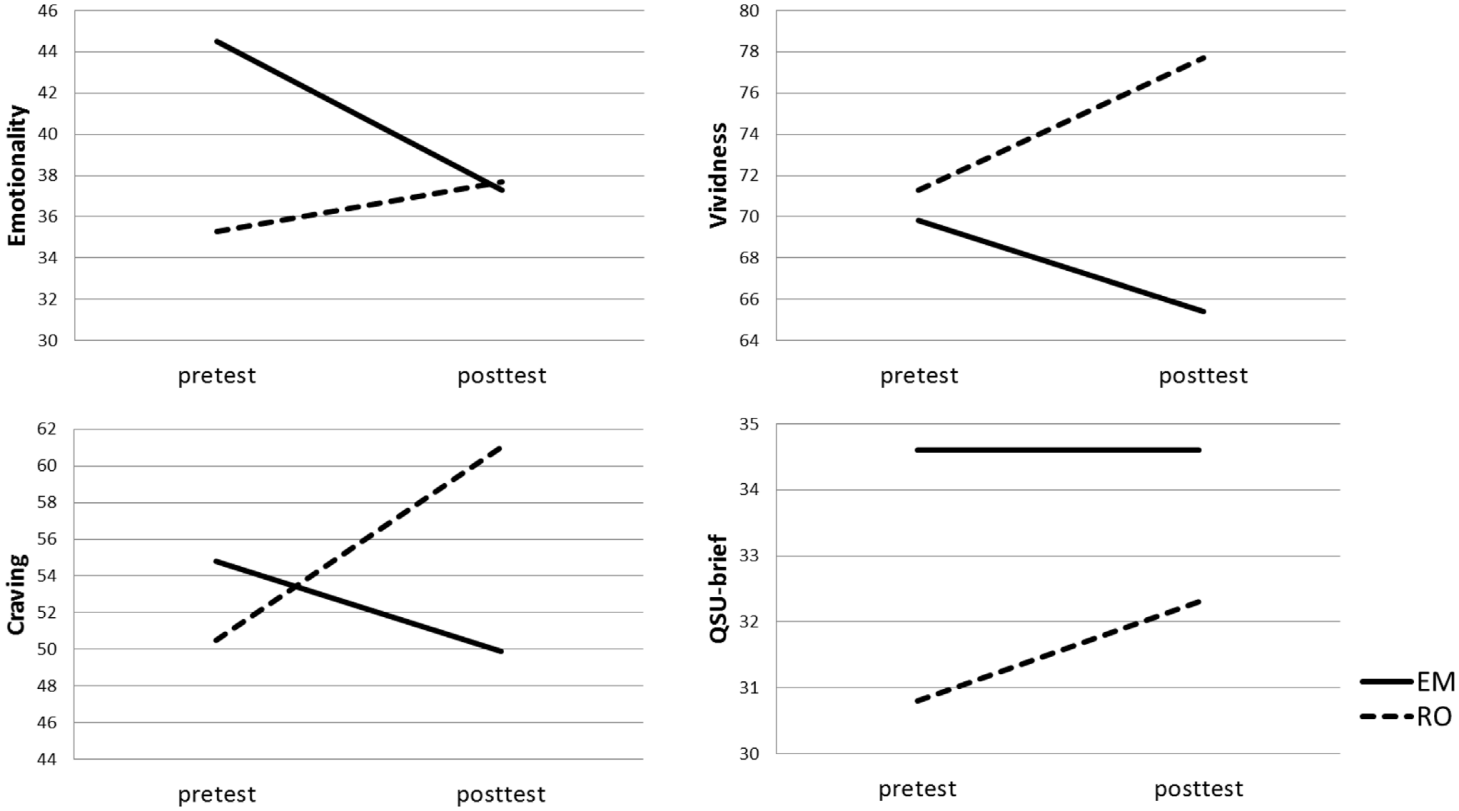
**Mean cigarette craving, emotionality, vividness, and Brief Questionnaire on Smoking Urges (QSU-brief) scores per Condition [recall + eye movements (EM), recall only (RO)] at pre- and posttest**.

Both in study 1 and 2, intervention effects were partially driven by vividness and craving increments in the RO condition (see also Figures 1 and 3). However, observing post-intervention dissociations between EM and RO after only four or six sessions of 24 s, while craving naturalistically increases during abstinence (60), and even more after active imagery (32, 34), is still clinically relevant. Because craving increases over time and/or due to imagery, one might assume that *if* craving would be increased to a maximum level prior to the experiment, an EM intervention would reduce craving. In the second study, we used a small craving induction procedure. Although craving significantly increased, the mean QSU-brief score after the craving induction was still only 3.3 (SD = 1.5), which is, on a scale from 1 to 7, definitely not maximal. Future studies should employ more thorough craving induction procedures to maximize craving levels at the start of the experiment.

Not all follow-up tests reached statistical significance. This might be explained by the fact that participants did not select the most suitable target images and memories. In study 1, not all participants selected foods that are typically craved (67, 68). In fact, only 47.2% selected high-caloric, sweet, or fatty foods (e.g., chocolate, cookies, fries, etc.). The other participants selected fruit, vegetables, and lunch or dinner meals. These more “neutral” foods are probably less sensitive to the EM intervention (69). In study 2, participants selected memories of specific, recent situations, and emotional states during which they experienced craving and smoked a cigarette. However, at pretest, image-specific craving scores were only 43.0 (SD = 30.8) on a scale ranging from 0 to 100. These relatively low pre-intervention craving scores might have prevented more substantial effects of EM and might explain why effects did not generalize to the more general craving measure (QSU-brief) at the end of the session. Also, because of these low craving scores, it is unclear how relevant these recent smoking memories actually are to smoking dependence. Memories of craving instances further back in the past might have a larger impact on current craving and smoking behavior. In future studies, an effort should be made to find out what specific memories contribute to current craving in each participant, and these memories should be targeted during the intervention. Other mental representations might serve suitable targets as well, such as trigger situations that someone is confronted with daily or weekly (e.g., waiting at a bus stop), associations of substance use with extremely pleasurable memories (e.g., first shot), or memories of prior relapse and loss of control (43).

In contrast to our hypothesis, dieting participants did not exhibit larger decreases in craving or image vividness after EM. This is in line with results from Kemps et al. (33) showing that watching dynamic visual noise during food imagery reduced image vividness and craving in both dieting participants and non-dieting controls. In the present study, however, there was a trend for dieters to show reduced intervention effects compared to non-dieters. This unpredicted finding might, however, be explained by their selection of food-related images: 33.3% chose a fruit or vegetable as their target food, compared to 12.8% in non-dieters group. As noted before, these foods are not typically craved, and more “neutral” targets have been observed to be less sensitive to recall + EM (69).

Furthermore, no spontaneous generalization effects were found for recall + EM on craving for non-recalled favorite foods, indicating that making EM during the imagination of one favorite food does not simply cause any other favorite food to become less desired. However, this finding might be explained by current methodology: non-targets were not explicitly retrieved prior to craving scorings, which might have prevented elaboration upon craving-related thoughts. Pretest craving scores were indeed lower for non-target foods (*M* = 66.9, SD = 15.2) than for target foods (*M* = 80.7, SD = 14.0).

In sum, despite non-maximal craving levels at the start of the experiment, and despite the fact that suboptimal target images were selected, we found significant effects of EM on the sensory richness of substance-related memories and imagery, associated craving, and subsequent behavior in two non-clinical samples. In line with previous studies, these data suggest that EM can be used as coping skill to temporarily reduce the intensity of craving. It remains to be investigated if EM can definitely alter substance-related memory and serve to reduce the occurrence or intensity of future cravings, without simultaneous taxing of WM. However, as noted before, several studies that adopted a similar design, i.e., the EMDR lab model, did observe effects that lasted over time [e.g., Ref. (22)].

It seems implausible that very short recall + EM interventions of the type used here will result in therapeutic effects. Note that in EMDR for PTSD, a series of sessions lasting 1 h or more are used to reduce the intensity and occurrence of trauma related flashbacks outside the clinic. It would be fascinating to test if the full EMDR procedure for food or drug craving may decrease craving in the long run and reduce relapse rates. However, it should first be established which images should best be targeted (memories, imagery, associations, cues, etc.), whether the effects are observed for all facets of craving [reward, relief, obsessive craving, see Ref. (70)], whether the effects of EM generalize to actual substance use behavior, and whether they generalize to people trying to control or quit their substance use, i.e., the eventual target group for the EMDR intervention.

## Author Contributions

ML: designed the experiments and supervised students during data collection, analyzed the data, and wrote the paper. MH and IE: provided valuable feedback.

## Conflict of Interest Statement

The authors declare that the research was conducted in the absence of any commercial or financial relationships that could be construed as a potential conflict of interest.

## Footnotes

^1^More specifically, EM targeted images of memories, cf., the EMDR protocol. See Section “Procedure” for more detailed information. To avoid confusion with the images formed in study 1, we will describe the images of study 2 as “memories.”

^2^An exploratory differentiation was made between the two types of memories, but no significant differences were observed. For the sake of comprehensiveness, we confine ourselves to the variables of primary concern.
